# Measuring similarity between gene interaction profiles

**DOI:** 10.1186/s12859-019-3024-x

**Published:** 2019-08-22

**Authors:** Joëlle Barido-Sottani, Samuel D. Chapman, Evsey Kosman, Arcady R. Mushegian

**Affiliations:** 1 Stowers Institute for Medical Research, Kansas City, MO USA; 2 École Polytechnique, Route de Saclay, Palaiseau, France; 3 Institute for Cereal Crops Improvement, School of Plant Sciences and Food Security, George S. Wise Faculty of Life Sciences, Tel Aviv University, Tel Aviv, Israel; 4 Department of Microbiology, Molecular Genetics and Immunology, Kansas University Medical Center, Kansas City, Kansas USA; 5 Present Address: Department of Ecology, Evolution and Organismal Biology, Iowa State University, Ames, Iowa USA; 6 Present Address: Booz Allen Hamilton, McLean, Virginia USA; 7 Present Address: Division of Molecular and Cellular Biosciences, National Science Foundation, Alexandria, Virginia USA

**Keywords:** Genetic interactions, Gene networks, Similarity measures, Slp1, SUN domain

## Abstract

**Background:**

Gene and protein interaction data are often represented as interaction networks, where nodes stand for genes or gene products and each edge stands for a relationship between a pair of gene nodes. Commonly, that relationship within a pair is specified by high similarity between profiles (vectors) of experimentally defined interactions of each of the two genes with all other genes in the genome; only gene pairs that interact with similar sets of genes are linked by an edge in the network. The tight groups of genes/gene products that work together in a cell can be discovered by the analysis of those complex networks.

**Results:**

We show that the choice of the similarity measure between pairs of gene vectors impacts the properties of networks and of gene modules detected within them. We re-analyzed well-studied data on yeast genetic interactions, constructed four genetic networks using four different similarity measures, and detected gene modules in each network using the same algorithm. The four networks induced different numbers of putative functional gene modules, and each similarity measure induced some unique modules. In an example of a putative functional connection suggested by comparing genetic interaction vectors, we predict a link between SUN-domain proteins and protein glycosylation in the endoplasmic reticulum.

**Conclusions:**

The discovery of molecular modules in genetic networks is sensitive to the way of measuring similarity between profiles of gene interactions in a cell. In the absence of a formal way to choose the “best” measure, it is advisable to explore the measures with different mathematical properties, which may identify different sets of connections between genes.

**Electronic supplementary material:**

The online version of this article (10.1186/s12859-019-3024-x) contains supplementary material, which is available to authorized users.

## Background

The results of genome-scale experiments often can be presented in the form of a matrix that describes quantitative behavior of genes in a specific measurement space. Frequently, the matrix is set up so that the rows represent genes or their products, the columns represent various conditions under which the properties of genes/gene products are assayed, and each matrix element is a numeric measurement associated with a gene in a particular condition. For instance, a matrix can characterize the gene expression space, where each column stands for the amount of specific mRNA present in a sample at a given time point, or under a specific drug treatment, or in a particular tissue in a multicellular organism. In all these cases, a matrix row consists of ordered measurements describing the transcript accumulation under the set of conditions, i.e., it can be viewed as a *gene expression vector*.

Other measurement spaces include, for example, protein-protein interaction space, where the data matrix consists of rows that may represent protein baits, and columns may represent, for example, purification samples; then, each matrix element is an event of product detection, or a measurement of its abundance, in a sample baited by a given protein, and the row corresponding to each gene product can be viewed as a *protein interaction vector*. A measurement space summarizing protein localization data may also be envisaged, where the columns are the defined locales in a cell, the matrix elements are the presences or intensities of protein reporter readouts at these locales, and each row is a *protein localization vector*.

In this study, we are concerned with the genome-wide vectors of yet another kind, i.e., *genetic interaction vectors*, which describe synthetic interactions of a null allele of a given gene with the null alleles of other genes in the same genome. In a genetic interaction matrix, both rows and columns correspond to genes (typically, those that are non-essential when deleted individually), and the matrix elements represent measurements of viability or fitness of the strain in which both genes are deleted.

Many research problems in genome sciences and in systems biology can be cast as the analysis of relationships between gene vectors, and a standard way to analyze these relationships is to find groups of gene vectors that are close to each other in a given measurement space. Many problems inherent in finding groups in a multidimensional measurement space has been explored (see the overviews of general issues, e.g. in [1–3]), and the need for defining tight groups of genes on the basis of their properties assessed at the genome scale have led to (re)invention of many cluster analysis methods by biologists [4–10].

**Fig. 1:**
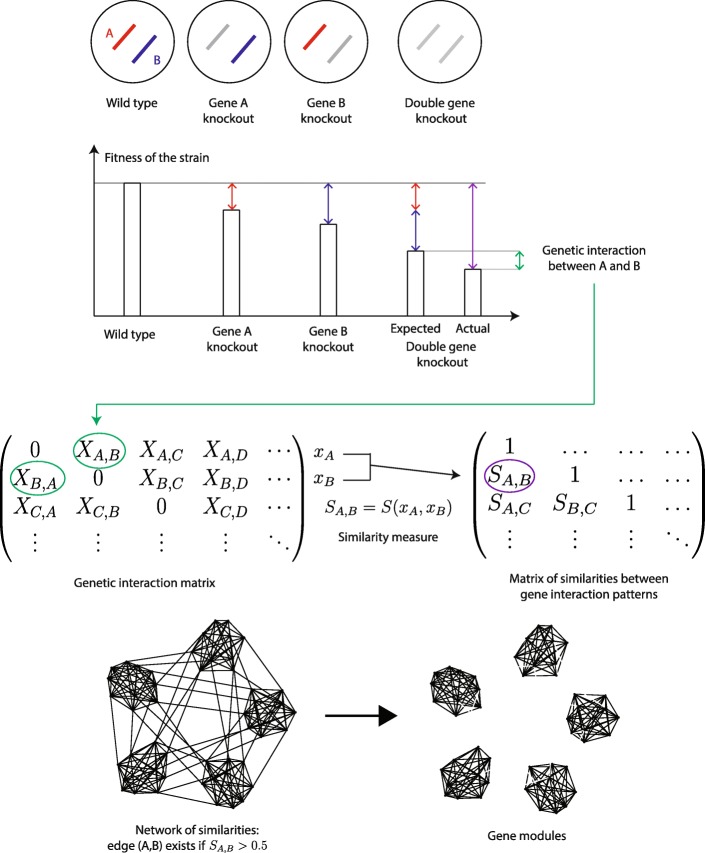
General outline of genetic interaction assays and schematics of the data transformations used in the process of their analysis

The representation of genomic data as complex networks is also popular (comprehensive discussion in [11]). In gene and protein networks, nodes typically represent genes or their products, and edges may link the pairs of genes that have a “biologically interesting” relationship. Sometimes such a relationship is a direct physical connection or interaction between two genes or their products, but, at least as often, the relationship is defined as similarity between the patterns of interaction of each gene with other molecules in the cell. For example, in many derivations of gene expression networks, an edge stands for a similarity in mRNA levels of two genes across many tested conditions, and not necessarily for a direct effect of one gene on the expression of the other. In protein interaction networks, an edge between two protein nodes may represent the similarity between the sets of purification partners for both proteins, rather than a direct contact between the two proteins. And in gene interaction networks, the edges may connect genes that have similar profiles of synthetic interactions, such as sickness or lethality, with other genes, regardless of the direct evidence of genetic interaction between a given pair (Fig. 1). The ability to use the genome-wide data to infer such links between genes, including the cases when one or both of the linked genes are otherwise uncharacterized, is one of the strengths of the systems approach.

Thus, a close connection exists between gene vectors and genome-wide networks: in many networks, an edge, by definition, is a link between a pair of genes whose genome-wide interaction vectors are highly similar. To reiterate, a gene interaction vector directly encodes experimentally detected interactions of a gene, whereas a gene network encodes gene relationships, which also may be called “gene interactions” in the literature. However, unlike the interactions directly recorded in a gene vector, the interactions in the latter sense are inferred from the properties of gene vectors. It may be useful to call the former “experimental interactions” and the latter “inferred interactions” to maintain awareness of the difference between the two.

The inferred interactions are the basis of many gene network representations, and they are often used for detecting modules in gene networks. Intuitively, a module is a set of genes that tend to have more experimental and/or inferred interaction events with each other, or perhaps stronger interactions, than is predicted by a particular random-interaction model; genes within a module also tend to have fewer or weaker interactions with genes outside the group than what is predicted by the model [12]. Thus, both definition and practical detection of a gene module are dependent, first, on the measure of closeness between genes and, second, on the choice of statistic comparing gene closeness to some expectation of closeness of random pairs of genes (Fig. 1).

This study focuses on one aspect of the analysis of the genetic interaction networks, namely the ways to measure the closeness between vectors of experimental interactions. The dataset that we have chosen for analysis has been produced using the Synthetic Genetic Array (SGA) family of approaches, which is based on the systematic screening of viability of double mutants [13–16]. In the best-studied setting, a viable strain of baker’s yeast *Saccharomyces cerevisiae* with a deleted non-essential query gene is crossed to an array of all other viable strains with single gene deletions. By comparing the fitness defect of a double mutant to the fitness defects in each of the two parents with single-gene deletions, one can measure the strength of interaction between these two genes [16–18].

In the foundational study (ref. [14]), genetic interaction networks were established from the interaction scores through a multistage algorithm that included several heuristic steps. Our study revisits the primary matrix of genetic interaction scores, applies different measures of closeness between the pairs of row vectors, and derives a secondary matrix, where the elements represent the degree of similarity between pairs of vectors (Fig. 1). We constructed similarity matrices using different measures of closeness between genetic interaction vectors and built the networks of genetic interactions on the basis of pairwise similarities of vectors rows in each matrix. We analyzed the properties of the resulting networks, their modular structure and the utility of induced modules for making biological inferences about gene function. Our main conclusion is that different similarity measures produce genetic interaction networks with different global properties and induce different gene modules in these networks.

## Results

### Clustered graphs and modules within them: different similarity measures result in different summary statistics for networks and modules

The SGA analysis defined gene interaction modules by a heuristic algorithm that employed a pairwise similarity measure between gene interaction vectors. The measure is based on Pearson correlation coefficient, but the algorithm uses many computational steps and employs extra information about gene function from the databases [14, 16, 18, 19]. We were interested in comparing this de facto standard with the performance of other measures of closeness, in particular those that may have mathematical properties distinct from Pearson correlation. To that end, we selected three other similarity measures, all of which operate on vectors with binary coordinates. The first reason for such relatively impressionistic choice was that the similarity measures of that type have mathematical properties different from the correlation-based measures. The second reason was that binary vectors and measures defined for them have been advocated for analysis of the genome-wide datasets in the literature, in part because continuous measurements are not always possible or may have to be discretized because of the technical concerns. The third reason was that some of the measures that we employed have been developed to correct certain undesirable properties of the measures applied to the analysis of genomic data previously (see also the Methods section).

**Fig. 2:**
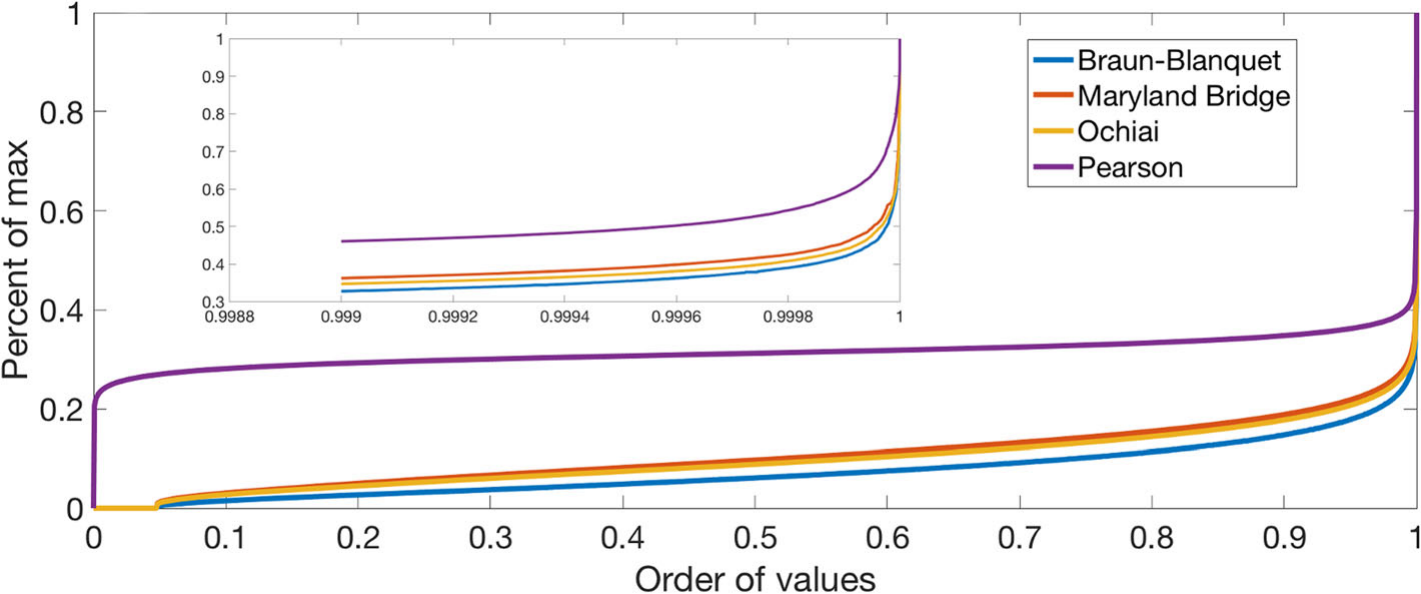
Cumulative similarity distributions between genetic interaction vectors under different similarity measures for the “one-square” transformation

The descriptive statistics for each distance measure after one kind of the data transformations that we used, i.e., the “one-square” transformation (see Methods) is presented in Table 1, with distance distributions for the vectors shown in Fig. 2. The summary statistics and distance distributions for the “two-squares” method (Additional file 1: Table S1 and Additional file 2: Figure S1, respectively) are deposited at the Zenodo data repository under the accession number 3361844, as indicated in the Availability of data and materials Section. Additional supporting information placed in the repository includes original interaction score matrices for the dataset, the eight distance matrices representing the four distances calculated for both the “one-square” and “two-squares” methods, and the lists of genes included in the matrices.

**Table 1:** Statistics of similarity scores between yeast genetic interaction vectors under different similarity measures for the one-square matrix

	Braun-Blanquet	Maryland Bridge	Ochiai	Pearson
Mean	0.04	0.06	0.06	< 0.01
Variance	0.01	< 0.01	< 0.01	< 0.01
Median	0.03	0.06	0.05	< 0.01
Minimum	0	0	0	−0.36
Maximum	0.53	0.60	0.57	0.81

The measure of similarity between vectors appears to have considerable effect on the shape of the distribution curves for the similarities between vectors. The Maryland, Ochiai, and Braun-Blanquet spaces are dominated by low similarity values; 99% of all distances are less than 0.2. In contrast, the curve for Pearson-based distances has the inflexion point near the 50th percentile of the data (Fig. 2). Thus, the choice of similarity measure may have an impact on the proportion of similarities that are registered as relatively high.

For each interaction matrix obtained with a particular similarity measure, we produced a network in which genes are represented as nodes and the weight of an edge represents the similarity score between the two genes it connects. In order to make the networks relatively sparse and more amenable to module analysis, we applied a weight threshold to each matrix, so that the 20,000 edges with the highest weight were retained, and the rest were removed. The selection of the network size at this step is arbitrary; there were typically around 2.10^7^ non-zero interactions in the square matrix, so 20,000 edges represented only a small fraction of all interactions, and the sets of nodes retained after this filtering may not be the same in all networks. The filtered networks sometimes contained small unconnected parts along with the main component. These sections were discarded, even though some of them may contain groups of functionally linked genes.

**Fig. 3:**
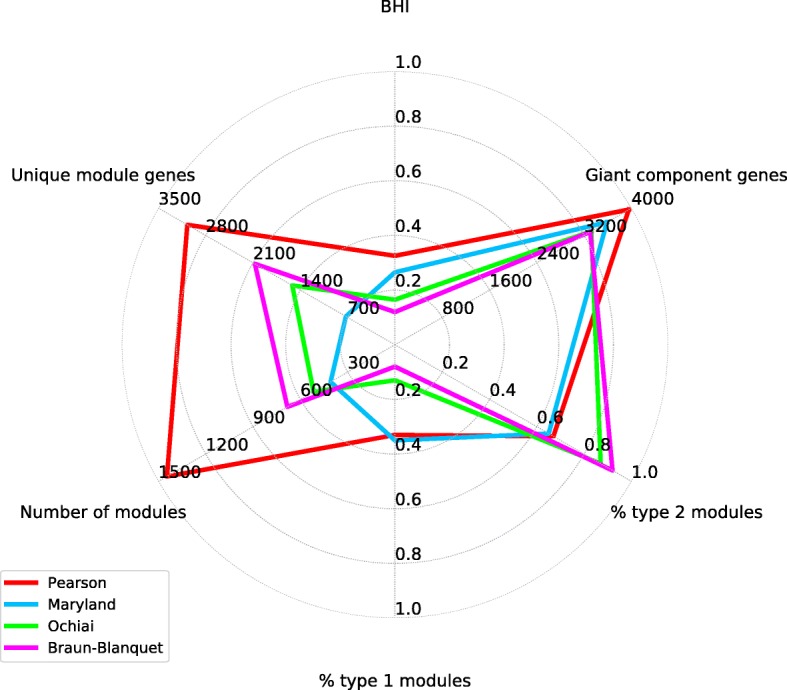
Select statistics of clustering and module annotation. The data are taken from Table 2

Some properties of the filtered networks are shown in Table 2 and Fig. 3. Unlike what was seen with the distribution of pairwise similarities between gene interaction vectors, there is no sharp difference between the networks induced by the binary vector-based similarities (Maryland bridge, Ochiai and Braun-Blanquet, referred to as M, O and B in the rest of the article) and the network built using Pearson correlation-based similarity (“P”). The latter has the largest number of nodes and the largest central connected component, but the difference in the number of nodes between the networks is not dramatic: the intermediate-sized network M contains ~ 10% fewer nodes than P, and O and B each contain 10% fewer nodes than M.

**Table 2:** Properties of gene interaction networks and modules derived from the networks under different similarity measures. All values are for the one-square matrix transformation method. See Methods and Discussion for detailed discussion, Figs. 3 and 4 for visual representation of the data, and supplementary online materials for generally similar results obtained under the two-square transformation

Similarity measure	Braun-Blanquet	Maryland Bridge	Ochiai	Pearson
Similarity threshold applied to retain ~ 20,000 edges in the network	0.16	0.18	0.20	0.15
Nodes (genes) in the network / nodes (genes) in the giant connected component	3427 / 3303	3610 / 3587	3385 / 3321	4038 / 3956
Edges in the network / edges in the giant connected component	20,020 / 19,943	20,065 / 20,052	20,067 / 20,032	20,016 / 19,967
Unique genes in modules / percentage of all genes in respective giant connected component	2072 / 62.7	725 / 20.2	1519 / 45.7	3072 / 77.6
Number of modules / unique genes per module	682 / 3.04	408 / 1.78	516 / 2.94	1446 / 2.12
Biological Homogeneity Index	0.12	0.27	0.16	0.33
Percentage of uncharacterized genes / *p*-value / clusters with uncharacterized genes	36 / 10^−38^ / 38	17 / 10^−11^ / 2	36 / 10^− 22^ / 30	26 / 10^− 3^ / 35
Annotated modules of Type 1 / Type 2	36 / 409	64 / 119	48 / 309	279 / 568

To find groups of genes with similar properties within these complex networks, we partitioned each of the clustering solutions with the aid of the widely used Girvan-Newman module-finding algorithm (ref. [12]). The algorithm has no intrinsic stopping rule and iterates until every edge is removed. We assumed that many functional gene modules are likely to contain between 5 and 50 nodes, and recorded all modules that fell within this range at any step of the recursive edge removal. A union of all such modules found in each network is called a “clustering” or a “clustering solution” below. A clustering is not a proper partition of the gene set, as some genes do not belong to any cluster, and the modules within a clustering may be nested.

Table 2 also shows the number of individual clusters and the number of genes appearing in at least one cluster for each clustering solution. As can be seen, different similarity measures between interaction vectors generated a different number of network modules. All clustering solutions included only a subset of all genes from the data, but, interestingly, the fraction of genes included in clusters differed between the clustering solutions in a way not readily predictable from the initial network size (Table 2 and Fig. 3). For the largest network P, 69% of proteins remained in clusters after stopping the algorithm; for the intermediate-sized M, only 16% of proteins remained in clusters; and the smallest networks O and B produced clusters comprising larger proportion, as well as larger absolute number of genes, than in the case of M.

**Fig. 4:**
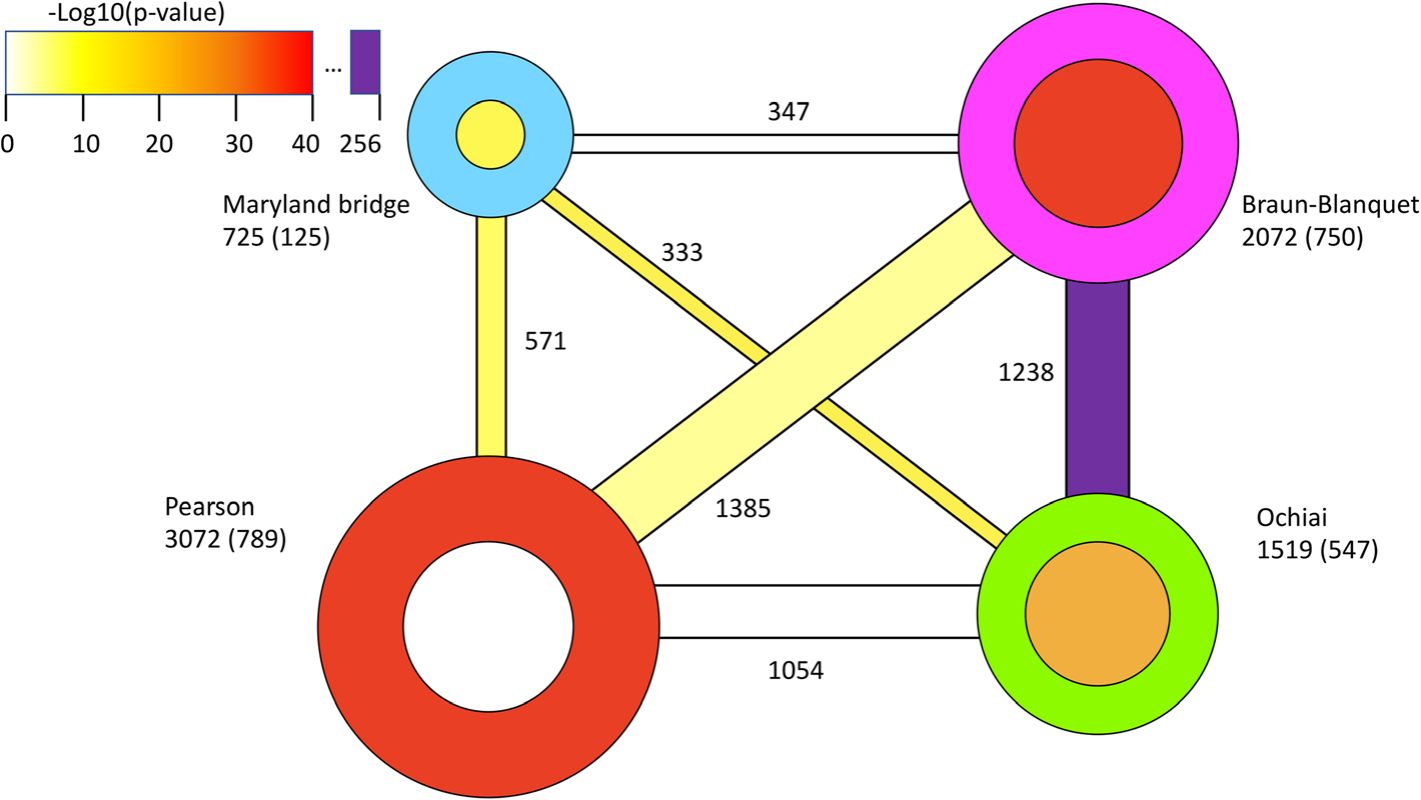
Genes shared between clustering solutions and the number of uncharacterized genes in each clustering solution. Line thicknesses represent genes shared by each pair of solutions, with the width proportional to their number, also shown next to each line. The band color represents the *p*-value of the number of shared genes between each pair of clusterings. The area of each circle is proportional to the number of genes shown next to the circle, and the size of each inner circle indicates the number of uncharacterized genes, shown in parentheses

Given the difference in the number of nodes in each of clustering solutions, we asked how the clusterings obtained from each network differed from each other. To that end, we computed the Clustering Error (CE) index for each pair of clusterings (see Methods). The values for the pair M and B, and well as pairs of P with each of the three other clustering, were between 0.91 and 0.93, whereas CE index for the B and O was 0.75, and for M and O it was 0.87, suggesting that some amount of non-redundant information may be present in different clustering solutions. Direct comparison of the shared and unique genes between the clustering solutions is shown in Fig. 4. The high number of shared genes in the Braun-Blanquet and Ochiai clusterings is particularly notable.

### All similarity measures induce modules with considerable functional information

The original analysis of the SGA data (ref. [14]) used a multistage, knowledge-based algorithm to divide their network into eight subnetworks, further splitting them into functional modules of various sizes. The approach was validated by the recovery of many known functional modules and definition of novel components of these modules. We were interested in whether our much simpler, parameter-poor approach could nevertheless approximate these results. We call a module from one of our clustering solutions *consistent* if it fulfills two separate conditions: i. 80% of the genes in the module have been assigned to one and the same cluster in [14], and ii. the proportion of the genes shared the same database annotation was at least 80% for clusters with 10 genes or more, or at least 50% for clusters with less than 10 genes. The clustering solution P is the closest to the original findings in [14], with about 26% of our modules mapping to at least one of the clusters characterized in that study (see Additional file 4: Table S3 in the Zenodo repository accession number 3361844, as indicated in the “Availability of data and materials” section, for the percentage of mapped modules for the four distance measures for both the “one-square” and “two-squares” methods. Descriptive summaries of the “two-squares” modules and networks, equivalent to the “one-square” Table 2, can also be found in Additional file 3: Table S2).

This result is of course expected, because the original module definition algorithm had used Pearson correlation for assessing similarity between interaction profiles. Other clusterings generated fewer consistent modules, but, as will be shown below, they may contain useful information not found in solution P.

We found that the proportion of uncharacterized genes included into modules is also different among clustering solutions (Table 2 and Fig. 4). The proportions of unknown genes in different clustering solutions were between 17 and 36% (average 27%). Statistical analysis, using Fisher’s exact test, indicates strong statistical overrepresentation of uncharacterized genes in clustering solutions B and O, and strong underrepresentation of uncharacterized genes in M (Table 2; see additional online information for the details of the calculation). This and other tests, discussed in the additional online information, suggest again that different distance measures induce modules that are not randomly drawn from the population, but may contain information about gene function not recovered by other measures.

We then examined all modules that contained between 5 and 11 genes. We called them Type 1 or Type 2 depending on whether, respectively, more or less than 50% of the genes in the cluster were already known to be functionally linked. Obviously, any such number is the lower bound, because some of the genes not known to have a connection to the rest of the module may in fact be so connected. The results, shown in Table 2 and Fig. 3, suggest that the clustering P produced the dramatically largest number of biologically characterized modules, with the largest number of genes included into them. The clustering B, in contrast, has eight times fewer biologically characterized modules.

Taken together, the data discussed in this as well as the previous section and visualized in Figs. 3 and 4 suggest that there are many similarities between clustering solutions B and O, and also considerable differences between those two solutions, as well as between them and solutions P and M. Our data statistics, as well as many specific examples, one of which is discussed in the following section, also suggests that modules from large and small networks overlap incompletely, i.e., clustering solutions from smaller networks are not all subsets of those from the larger networks.

### Novel putative connections between genes: SUN domains may be associated with protein glycosylation

The SUN (Sad1-UNC-84 homology) domains are present in all eukaryotes, typically in proteins that are associated with the nuclear envelope and play roles in nuclear migration, meiotic telomere tethering, and other processes related to nuclear dynamics; the role of SUN domains is incompletely defined but may involve mediation of protein-protein interactions in the perinuclear space (reviewed in [20]). The all-beta fold for the SUN domain family has been predicted, and distant sequence similarity of SUN domains to the carbohydrate-binding discoidin domain has been pointed out [21]. The subsequently determined three-dimensional structure of a human SUN2 protein confirmed the all-beta structure of the protein but did not address the carbohydrate connection [22]. A direct comparison of a SUN2 structure (pdb 3UNP) with a database of protein three-dimensional structures, however, reports discoidin as its best match, followed by various sugar-binding domains, often comprising the non-catalytic moieties of the bacterial and eukaryotic sugar-modifying enzymes, with convincing z-scores of 12–16 (searches performed in July 2018 using DALI web server [23]). This is compatible with a hypothesis that SUN domains interact with carbohydrates – most likely, with glycosylated proteins. Such interactions, however, have not been demonstrated experimentally.

Inspection of modules detected in this work shows that one SUN-domain protein in yeast, the product of the Slp1 gene, is found in module 638 of clustering P, together with several genes involved in protein glycosylation in the endoplasmic reticulum (ER). These genes are Alg3, Alg6, Alg12 and Die2, encoding glycosyltransferases that synthesize the dolychol-linked oligosaccharide and transfer it to the asparagine residues in the target proteins, as well as Spc2, a subunit of signal peptidase that cleaves a leader peptide off the proteins secreted via ER. Also in this cluster is an uncharacterized integral membrane protein YER140w/Emp65, which has been shown to interact genetically with Slp1 [24]; the complex of those two proteins in yeast apparently protects soluble proteins from degradation as they are delivered to the ER lumen [25]. Interestingly, in the module 347 of our smallest clustering solution B, yeast Slp1 is found together with yet another glycosyltransferase, cytoplasmic glycogenin Glg2, which primes glycogen synthesis by conjugating itself to a molecule of glucose that is then extended into a polysaccharide chain by glycogen synthase.

Protein glycosylation is thought to be sensed by the proteostasis machinery in ER, though it has been noted also that yeast Slp1-Emp65 complex protects glycosylated as well as non-glycosylated proteins [25]. On the other hand, a plant ortholog of Emp65, called POD1 in *A.thaliana*, is known to facilitate pollen tube guidance in response to micropylar female signaling [26], a pathway in which multiple components of protein glycosylation machinery in the ER are also involved [27, 28]. All things considered, it is worth investigating whether Slp1 and other SUN-domain proteins play a role in glycosylation of proteins delivered to the continuum of nuclear and ER lumen, in facilitating maturation and functions of glycosylated proteins in this compartment, or in interactions of the protein glycosylation machinery with other lumen components. Moreover, inferred genetic interactions of Slp1 and Glg2 may add support to the predicted connection of SUN domains to protein glycosylation, and perhaps suggest that glycogenins may have additional roles in yeast cell beyond priming glycogen synthesis.

## Discussion

In this work, we investigated one step in the process by which the units of molecular function in a eukaryotic cell – the modules of genes and their products that “work together” – are computationally defined. The property of “working together” is not always determined by a direct experimental measurement, but more often by inference. Such inferences from the raw data are done by a multi-stage analysis, ridden with assumptions and sensitive to the choices of algorithms, parameters and heuristics made along the way.

Mani et al. (ref. [17]) have highlighted the fact that a genetic interaction itself can be defined in many ways. For a pair of genes (x, y), given the values of the fitness phenotypes of the single mutants, *Wx*, *Wy*, one may be interested also in the value of the expected fitness phenotype of a double mutant *E*(*Wxy*). The value of *E*(*Wxy*) may be set to *min*(*Wx*, *Wy*), or to (*Wx* · *Wy*), or take some other form, and interaction between the pair of genes in each case can be defined as a significant deviation from *E*(*Wxy*). That study concluded that different definitions of interaction, when applied to the same raw mutant-fitness data set, may give either negatively or positively shifted distributions of the fitness values. Moreover, interaction networks inferred using different definitions of genetic interaction vary greatly in their quantitative properties and in the modules of functionally interacting proteins discovered in them.

Our work is similar in spirit, but we studied a different stage of network inference; our main focus was not on the comparison of the ways to obtain or transform the values of the elements in the interaction matrix, but rather on the measurement of similarity between ordered sets of these elements. Just as there are many ways to define genetic interaction, there are many possible measures of (dis)similarity between interaction vectors. There is an extensive literature on the mathematical and statistical properties of those measures, as well as on connections and differences between different measures [9, 29–33], but only a limited guidance exists on how to select a good way to assess (dis)similarity between vectors representing genome-scale data (see discussion in ref. [9]).

In our re-analysis of a well-studied set of yeast functional modules produced by the SGA platform, we asked two specific questions: first, whether it is possible to recover a significant portion of the known functional information using a simple approach to network edge definition and standard community detection algorithm, as long as Pearson-based similarity measure is employed; and second, whether the application of other kinds of similarity measures would produce similar or very different results, and whether some of the measures may lead to significant loss, or perhaps to partial gain, of signal in the data.

The results of some of our analyses were as expected, while others were more surprising. Expectedly, in nearly all tests, P-clustering and P-modules were the most informative, with the majority of the P-modules mapping to the already inferred functional units in the yeast cells, which were in the first place discovered by a multistep procedure based on a Pearson correlation-based similarity measure, verified against the known biology. Interestingly, if perhaps also not too surprisingly, the fraction of our P-modules mapped to the modules from the original study was substantial, despite the simplicity of our inference procedure. Thus, the answer to our first question is “Yes”: the bulk of state-of-the-art information on the functional modules can be obtained by employing a parameter-poor model and a generic algorithm to find communities in the network.

Less expected is the fact that the answer to our second question is also a qualified “Yes”: similarity measures applicable to the vectors with binary coordinates also recover considerable functional information, including non-redundant evidence of functional links between genes. It is also worth mentioning that at different steps of the analysis, the statistics comes out in unexpected ways for different similarity measures: for example, the M distance gives a much larger proportion of functional gene modules than O and B distances, whereas the latter two are the ones most enriched with functional links that were scored as novel in the manual re-annotation (Table 1, Figs. 3 and 4).

Our study is far from comprehensive; we have not discussed many similarity measures that have been proposed in the literature for the analysis of genomic data. It should be noted that the P similarity measure is defined for binary coordinates and interval coordinates alike, and that generalizations for the case of interval coordinates are available for M, O, B and many other similarity measures. Furthermore, the thresholding steps to limit the number of edges in a complex network, as well as converting weighted edges to unweighted ones, lead to post hoc data discretization, potentially with loss of sensitivity, and systematic analysis of these elements of the inference procedure should also be of interest.

In the recent years, several thorough comparisons of similarity measures for analysis of multidimensional data sets have been published. For example, Deshpande et al. [34] focused specifically on the effect of similarity measures on the properties of genetic interaction networks from different model systems, whereas Shirkhorshidi et al. [35] examined the effect of the choice of dissimilarity measure on the analysis of continuous data in several ‘big data’ sets, mostly from outside of biology. Extensive benchmarking in these studies revealed that the performance of a measure in recovering the known signal in the data is not always easy to explain on the basis of its mathematical properties, and that measures for vectors with binary and interval coordinates may recover non-identical subsets of the known relationships from the same data. Similar observations have been reported recently in the analysis of population structures that relied on genome-wide vectors with the coordinates represented by genetic markers, where the choice of presentation of the variables (categorical, transformed binary, or interval) and of different dissimilarity metrics affected the results in a significant way [36].

In an earlier work, it has been hypothesized that the shape of the distribution of the (dis)similarity measures for a particular dataset may be indicative of the performance of a measure in finding signals of interest in that dataset; at least in some cases, “good” measures are those for which the higher momenta of the distribution reach their extremes [9]. If this turns out to be a general rule, then it is possible that the optimal (dis)similarity measure depends on the structure and the idiosyncrasies of the dataset under study as much as on the mathematical properties of any specific measure.

## Conclusions

We re-analyzed well-studied data on yeast genetic interactions, asking whether the choice of the similarity measure between pairs of gene vectors may impacts the properties of gene interaction networks and of putative functional gene modules detected within them. The four networks and four sets of modules obtained in our study induced different numbers of putative functional gene modules, and each similarity measure induced some unique modules. It appears that different similarity measures, even those resulting in a small and relatively fragmented clustering solutions, may nevertheless provide missing or complementary information helpful for generation of biological hypotheses.

## Methods

### The dataset

The results of analysis of *S. cerevisiae* SGA [14] have been obtained from DRYGIN, the Data Repository for Yeast Genetic Interactions [37, 38]. The implementation of the SGA procedure used 1711 query genes, each of which was crossed with an array of 3885 deletion mutants. Some genes were tested under multiple conditions, such as different temperatures; in that case, each occurrence of a gene was treated as a separate gene. Let *N*_*query*_ represent the number of query genes (1711), *N*_*array*_ represent the number of array genes (3885), and *N*_*all*_ represent the number of distinct genes encountered among the query and array genes. Let the set of query genes be denoted by *G*_*query*_, the set of array genes be denoted by *G*_*array,*_ and the union of two sets be denoted by *G*_*all*_. Some genes are in both *G*_*query*_ and *G*_*array*_, such that the total number *N*_*all*_ of unique genes in *G*_*all*_ is 4457.

The initial matrix of interaction scores **X** is a rectangular matrix with dimensions 1711 by 3885, where rows are represented by *G*_*query*_ and columns by *G*_*array*_. In this matrix each element x_ij_ is the interaction score between query gene *i* and array gene *j*, so that the vector **v**_**i**_ = (*v*_*i1*_, …, *v*_*i3885*_) is the genetic interaction vector of query gene *i*. The element *v*_*ij*_ can be positive or negative or zero, depending on the kind of interaction between genes. One can also define the column vectors of array genes: **c**_**i**_ = (*v*_*1j*_, …, *v*_*1711j*_).

Our goal is to score the similarity of interaction profiles of each pair of genes with all their interaction partners. We calculated similarity scores in two ways. The first approach, the “two squares” method, was essentially the same as in ref. [14]. In this method, two similarity matrices **Q** and **A** were created from the original interaction matrix **X**.

Matrix **Q** was created by calculating similarity scores (distances) between every pair of the query vectors. **Q** was thus a symmetric matrix of size 1711 by 1711. That is,

$$ Q=\left[\begin{array}{cccc}{q}_{11}& {q}_{12}& \cdots & {q}_{1{N}_{query}}\\ {}{q}_{21}& {q}_{22}& \cdots & {q}_{2{N}_{query}}\\ {}\vdots & \vdots & \ddots & \vdots \\ {}{q}_{N_{query}1}& {q}_{N_{query}2}& \cdots & {q}_{N_{query}{N}_{query}}\end{array}\right] $$

and

$$ dist\left({v}_i,{v}_j\right)={q}_{ij}, $$

where *v*_*i*_ and *v*_*j*_ are row vectors of **X** and dist() is the value of an arbitrary distance measure (see below).

Matrix **A** was created using the same method as matrix **Q**, except that in this case, similarity scores were calculated between every pair of the array vectors (the columns of **X**). Therefore, **A** was a symmetric matrix of size 3885*3885:

$$ A=\left[\begin{array}{cccc}{a}_{11}& {a}_{12}& \cdots & {a}_{1{N}_{array}}\\ {}{a}_{21}& {a}_{22}& \cdots & {a}_{2{N}_{array}}\\ {}\vdots & \vdots & \ddots & \vdots \\ {}{a}_{N_{array}1}& {a}_{N_{array}2}& \cdots & {a}_{N_{array}{N}_{a\mathrm{r} ray}}\end{array}\right] $$

and

$$ dist\left({c}_i,{c}_j\right)={a}_{ij}. $$

The similarity scores in matrices **Q** and **A** were then placed in a 4457 by 4457 (*N*_*all*_ by *N*_*all*_) supermatrix, **S**. The rows of the supermatrix **S** are the elements of *G*_*all*_, and so are the columns of **S**.

Each element, ${Q}_{Gquery_i,{Gquery}_j}$ in **Q** that corresponds to the *ith* and *jth* query genes is mapped to the element in **S** that corresponds to the positions of these genes in *G*_*all*_: ${S}_{Gall_k,{Gall}_l}$. In other words:

$$ \forall \left(i,j\in {G}_{query}\right),{Q}_{Gquery_i,{Gquery}_j}\to {S}_{Gall_k,{Gall}_l},\left(k,l\in {G}_{all}\right) $$

The mapping for the elements in the **A** matrix, analogously, is:

$$ \forall \left(i,j\in {G}_{array}\right),{Q}_{Garray_i,{Garray}_j}\to {S}_{Gall_k,{Gall}_l},\left(k,l\in {G}_{all}\right) $$

For each element ${Q}_{Gquery_i,{Gquery}_j}$ mapped to **S**, if neither *G*_*queryi*_ nor *G*_*queryj*_ were also in *G*_*array*_, (or vice versa, if neither *G*_*arrayi*_ nor *G*_*arrayj*_ were also in *G*_*query*_), then the corresponding row or column is missing **S** and is ignored in the following. If both *G*_*queryi*_ and *G*_*queryj*_ were in *G*_*array*_, (or if both *G*_*arrayi*_ and *G*_*arrayj*_ were in *G*_*query*_) then the element in **S** was averaged with the corresponding element in **A** for those genes. Otherwise, the value was unchanged. Because of this procedure, the resulting supermatrix was symmetrical.

The second method used to calculate similarity scores, the “one square” method, first places all elements from the *N*_*query*_ by *N*_*array*_ matrix **X** into their corresponding elements of a supermatrix **R** (a square matrix of the size *N*_*all*_ by *N*_*all*_, i.e., 4457 by 4457) which only has interaction scores. That is,

$$ \forall \left(i\in {G}_{query},j\in {G}_{array}\right),{X}_{Gquery_i,{Garray}_j}\to {R}_{Gall_k,{Gall}_l},\left(k,l\in {G}_{all}\right) $$

If no interaction experiment of the pair of genes in a particular ${R}_{Gall_k,{Gall}_l}$ element had been performed, then that element in **R** was set to zero. If both a query-array and array-query experiment had been performed on the ${R}_{Gall_k,{Gall}_l}$ element (that is, if both genes were both in *G*_*query*_ and *G*_*array*_), then that element in **R** was averaged between the two values. Otherwise, the value stayed the same. After this process, the resulting supermatrix was symmetrical.

(Dis)similarity scores were calculated on the pairs of rows of the supermatrix **R**, creating a symmetric supermatrix **S**. Denoting rows (row vectors) of **R** as *v*,

$$ dist\left({v}_i,{v}_j\right)={S}_{ij} $$

The data transformed by these two methods were then analyzed as described in the rest of the Methods section, and descriptive statistics was collected at several stages. The data transformed by these two methods were then analyzed as described in the rest of the Methods section. The results for both methods were similar (Table 1 and Fig. 2 show, respectively, distance matrix summary statistics and distance distributions for the “one-square” transformation; equivalent data for the “two-squares” transformation are available at Zenodo, accession number 3361844).

### Similarity/dissimilarity between vectors

Many measures of (dis)similarity between vectors have been proposed in the literature. We now describe several measures employed in this study and discuss some of their notable properties. In the following, *X* · *Y* is the dot (inner) product of two vectors ***X*** and ***Y***, and $\left\Vert X\right\Vert =\sqrt{X\cdotp X}$.

**Maryland bridge** (*Mb*) coefficient of similarity, proposed in [39], is defined for binary vectors ***X*** and ***Y*** as

1 $$ \mathrm{Mb}\left(\mathrm{X},\mathrm{Y}\right)=\frac{1}{2}\left(\frac{X\cdotp Y}{{\left\Vert X\right\Vert}^2}+\frac{X\cdotp Y}{{\left\Vert Y\right\Vert}^2}\right),\kern0.5em $$

*Mb* can take values from zero for a pair of vectors that do not share 1 s at any position, to one for any pair of identical non-zero vectors. Higher score means higher similarity. For two vectors of same length that share half of their 1 s, their *Mb* is 0.5; such property is also observed with the Dice (Sørensen-Dice) coefficient, given by the eq. (2), whereas better-known Jaccard similarity coefficient provides a counter-intuitive value of 1/3 in such a case.

2 $$ D\left(\mathrm{X},\mathrm{Y}\right)=2\frac{X\cdotp Y}{{\left\Vert X\right\Vert}^2{\left\Vert Y\right\Vert}^2},\kern0.75em $$

**Ochiai** (*O*) coefficient of similarity is defined for binary vectors ***X*** and ***Y*** as

3 $$ O\left(\mathrm{X},\mathrm{Y}\right)=\frac{X\cdotp Y}{\left\Vert X\right\Vert \left\Vert Y\right\Vert },\kern0.75em $$

The values of *O* can be from zero to one.

**Braun-Blanquet** (*BB*) similarity coefficients for binary vectors ***X*** and ***Y*** include

4 $$ {\mathrm{BB}}_{\mathrm{max}}\left(\mathrm{X},\mathrm{Y}\right)=\frac{X\cdotp Y}{\max \left({\left\Vert X\right\Vert}^2,{\left\Vert Y\right\Vert}^2\right)} $$

and

5 $$ {\mathrm{BB}}_{\mathrm{min}}\left(\mathrm{X},\mathrm{Y}\right)=\frac{X\cdotp Y}{\min \left({\left\Vert X\right\Vert}^2,{\left\Vert Y\right\Vert}^2\right)},\kern0.75em $$

They correspond the number of 1s shared by two vectors, normalized by the largest (eq. 5) or the smallest (eq. 6) number of 1s in either of the two vectors. Both coefficients of similarity range from zero to one, and a higher score means a higher similarity. Interestingly, the Dice similarity (eq. 2) equals the harmonic mean of the two Braun-Blanquet coefficients.

**Pearson correlation** (*r*) coefficient between two, possibly non-binary, vectors ***X*** and ***Y*** is given by the formula

6 $$ r\left(\mathrm{X},\mathrm{Y}\right)=\frac{X\cdotp Y-n\overline{x}\overline{y}}{\sqrt{\left({\left\Vert X\right\Vert}^2-n{\overline{x}}^2\right)\left({\left\Vert Y\right\Vert}^2-n{\overline{y}}^2\right)}}=\frac{\sum_{\mathrm{i}=1}^n\left({x}_i-\overline{x}\right)\left({y}_i-\overline{y}\right)}{\sqrt{\sum_{\mathrm{i}=1}^n{\left({x}_i-\overline{x}\right)}^2}\sqrt{\sum_{\mathrm{i}=1}^n{\left({y}_i-\overline{y}\right)}^2}}, $$

where $\overline{x}=\frac{1}{n}{\sum}_{\mathrm{i}=1}^n{x}_i$ and $\overline{y}=\frac{1}{n}{\sum}_{\mathrm{i}=1}^n{y}_i$. It ranges from − 1 to 1.

For the computation of the Pearson correlation coefficient, the raw gene interaction on the interval coordinates were used directly. In the case of other similarity measures, a threshold of 0.5 was applied to the raw interaction scores to transform them into binary data: the scores higher than the threshold were converted into a 1 and score lower than a threshold (including a handful of negative scores, all of which had small absolute values – see Table 1) into a 0.

### Network visualization, partitioning and cluster assessment

To detect clusters in the networks, the algorithm of Girvan and Newman [12] was applied, as implemented through the BGL toolbox [40] and Matlab release R2009b (distributed by Mathworks). The algorithm evaluates the centrality of each edge in the network by computing the shortest paths between each possible pair of nodes in the network; the shortest path is defined as such path between two nodes that minimizes the number of edges for unweighted graphs, or minimizes the sum of weights on the edges for weighted graphs. The algorithm counts how many such shortest paths include (“use”) each edge, and removes the most-used edge from the graph. The shortest paths are recalculated for the modified graph, in which the centrality of some edges may have changed.

The Clustering Error (CE) index [41] is defined as

7 $$ \mathrm{CE}\left(\mathrm{S},\mathrm{S}\hbox{'}\right)=\frac{\left|U\right|-D}{\left|U\right|},\kern0.75em $$

where |U| is the total number of elements in S and S′. *CE* measures the overlap between two clustering solutions S = {S_i_} and S′ = {S_j_’} from the confusion matrix M where m_i,j_ is the number of elements shared by S_i_ and S_j_’. This matrix is transformed with the Hungarian algorithm, which associates each cluster from S with one cluster from S′ in order to maximize the total number of shared elements between pairs of clusters, *D*. The resulting index ranges from zero for perfect identity of clustering solutions to one. The clustering error was calculated using M.Buehren’s Matlab package for the Hungarian algorithm [42].

To assess the biological plausibility of the gene modules, we used yeast gene ontology [43], focusing on the ‘biological process’ hierarchy. The biological homogeneity index (BHI; ref. [44]) of a cluster C is

8 $$ \mathrm{BHI}(C)=\frac{1}{n\left(n-1\right)}{\sum}_{x\ne y\in C}I\left(\mathrm{x},\mathrm{y}\right),\kern0.5em $$

where n is the number of annotated genes in the cluster and *I(x,y)* is equal to 1 if the genes x and y share at least one functional annotation, and 0 if not. This index represents the probability that two annotated genes found in the same cluster are functionally linked. It ranges from 0 to 1, with a higher score meaning a greater homogeneity. This index can also be applied to the entire clustering solution, and in that case is the average of the scores of all clusters in that clustering. The clusters for which a score could not be calculated are not considered in this average.

## Additional files

Additional file 1: **Table S1.** Statistics of similarity scores between yeast genetic interaction vectors under different similarity measures for the two-square matrix. (DOC 12 kb)

Additional file 2: **Figure S1.** Cumulative similarity distributions between genetic interaction vectors under different similarity measures for the two-square transformation. (PDF 179 kb)

## Data Availability

All data generated or analyzed during this study are included in this published article, its Additional files, or deposited at the Zenodo public depository, acc. 3361844 10.5281/zenodo.3361844, as well as Stowers Institute Original Data Repository (Stowers ODR), linked at https://www.stowers.org/research/publications.

## Abbreviations

BB: Braun-Blanquet dissimilarity measure and clustering solution
CE: Clustering Error
ER: endoplasmic reticulum
M and Mb: Maryland Bridge dissimilarity measure and clustering solution
O: Ochiai dissimilarity measure and clustering solution
P: Pearson correlation-based dissimilarity measure and clustering solution
SGA: Synthetic Genetic Array
