# Supplementary material for: Measuring similarity between gene interaction profiles
Source: BMC Bioinformatics. 2019 Aug 22;20:435. doi: 10.1186/s12859-019-3024-x (PMC6704681; doi:10.1186/s12859-019-3024-x)
Supplement: Supplementary file 1 — Table S1. Statistics of similarity scores between yeast genetic interaction vectors under different similarity measures for the two-square matrix. (DOC 12 kb) [file 12859_2019_3024_MOESM1_ESM.doc]

| Similarity measure | Mean | Variance | Median | Minimum | Maximum |
| --- | --- | --- | --- | --- | --- |
| Braun-Blanquet | 0.0519 | 0.0010 | 0.0472 | 0.0 | 0.5705 |
| Maryland bridge | 0.0776 | 0.0017 | 0.0746 | 0.0 | 0.6465 |
| Ochiai | 0.0708 | 0.0014 | 0.0681 | 0.0 | 0.6330 |
| Pearson | 0.0038 | 0.0018 | 0.0028 | -0.3563 | 0.8560 |
