# Supplementary figures and images for: Measuring similarity between gene interaction profiles
Source: BMC Bioinformatics. 2019 Aug 22;20:435. doi: 10.1186/s12859-019-3024-x (PMC6704681; doi:10.1186/s12859-019-3024-x)

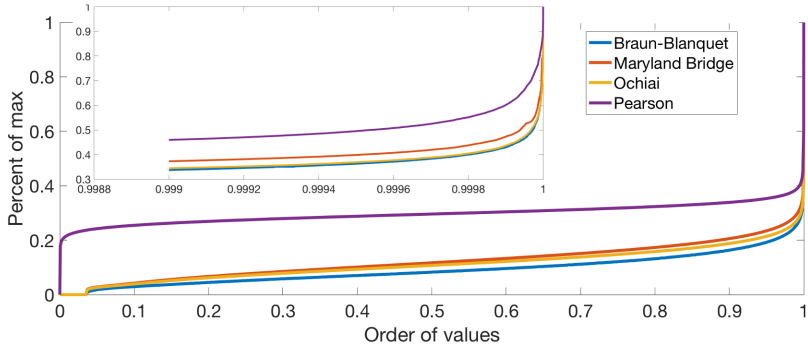

Supplement: Supplementary file 2 — Figure S1. Cumulative similarity distributions between genetic interaction vectors under different similarity measures for the two-square transformation. (PDF 179 kb) [file 12859_2019_3024_MOESM2_ESM.pdf]
